# Assessing the use of blood microRNA expression patterns for predictive diagnosis of myxomatous mitral valve disease in dogs

**DOI:** 10.3389/fvets.2024.1443847

**Published:** 2024-11-01

**Authors:** Javier Palarea-Albaladejo, Elizabeth F. Bode, Catheryn Partington, Mattia Basili, Elzbieta Mederska, Hannah Hodgkiss-Geere, Paul Capewell, Caroline Chauché, Robert M. Coultous, Eve Hanks, Joanna Dukes-McEwan

**Affiliations:** ^1^Department of Computer Science, Applied Mathematics and Statistics, University of Girona, Girona, Spain; ^2^Department of Small Animal Clinical Science, School of Veterinary Science, Leahurst Campus, University of Liverpool, Neston, United Kingdom; ^3^School of Molecular Biosciences, College of Medical, Veterinary and Life Sciences, University of Glasgow, Glasgow, United Kingdom; ^4^Centre for Inflammation Research, The Queen’s Medical Research Institute, University of Edinburgh, Edinburgh, United Kingdom; ^5^MI:RNA Ltd, Edinburgh, United Kingdom

**Keywords:** miRNA, biomarkers, predictive modeling, MMVD, chronic valvular disease, canine

## Abstract

**Background:**

Myxomatous mitral valve disease (MMVD) is a common, acquired, and progressive canine heart disease. The presence of heart murmur and current cardiac biomarkers are useful in MMVD cases but are not sufficiently discriminatory for staging an individual patient.

**Objectives:**

This study aimed to conduct a preliminary assessment of canine serum and plasma expression profiles of 15 selected miRNA markers for accurate discrimination between MMVD patients and healthy controls. Additionally, we aim to evaluate the effectiveness of this method in differentiating between pre-clinical (stage B1/B2) and clinical (stage C/D) MMVD patients.

**Animals:**

Client-owned dogs (*n* = 123) were recruited for the study. Following sample exclusions (*n* = 26), healthy controls (*n* = 50) and MMVD cases (*n* = 47) were analyzed.

**Methods:**

A multicenter, cross-sectional, prospective investigation was conducted. MicroRNA expression profiles were compared among dogs, and these profiles were used as input for predictive modeling. This approach aimed to distinguish between healthy controls and MMVD patients, as well as to achieve a more fine-grained differentiation between pre-clinical and clinical MMVD patients.

**Results:**

Performance metrics revealed a compelling ability of the method to differentiate healthy controls from dogs with MMVD (sensitivity 0.85; specificity 0.82; and accuracy 0.83). For the discrimination between the pre-clinical (*n* = 29) and clinical (*n* = 18) MMVD cases, the results were promising (sensitivity 0.61; specificity 0.79; and accuracy 0.73).

**Conclusion and clinical importance:**

The use of miRNA expression profiles in combination with customized probabilistic predictive modeling shows good scope to devise a reliable diagnostic tool to distinguish healthy controls from MMVD cases (stages B1 to D). Investigation into the ability to discriminate between the pre-clinical and clinical MMVD cases using the same method yielded promising early results, which could be further enhanced with data from an increased study population.

## 1 Introduction

Myxomatous mitral valve disease (MMVD) is the most common cardiovascular disease in dogs (1, 2), with greater prevalence in smaller dogs (<20 kg) and marked age-related occurrence, with up to 85% of dogs having valvular lesions by the age of 13 years (1, 3). Progressive degenerative mitral valve lesions lead to mitral regurgitation (associated with the presence of a systolic heart murmur on auscultation). As mitral regurgitation progresses, it results in gradually increasing left-sided volume overload and eventually left-sided filling pressures, which may eventually result in left-sided congestive heart failure (CHF) (3). MMVD can be graded in four stages: stage A, healthy dogs at risk of disease; stage B, dogs with evidence of mitral valve regurgitation but no clinical signs of CHF; stage C, dogs with clinical signs of CHF; or stage D, dogs with clinical signs of CHF refractory to treatment (4). Stage B, the pre-clinical period, is subdivided into stage B1 for patients with no significant cardiac remodeling, and stage B2 when there is evidence of left atrial and left ventricular enlargement (4).

Serial echocardiographic examination is recommended as the most sensitive method of confirming the diagnosis, staging, and monitoring of MMVD (4), but is not always available in a clinical setting. Concentrations of blood cardiac biomarkers (CBs), such as N-terminal pro-brain natriuretic peptide (NT-proBNP) and cardiac troponin I (cTnI), can be clinically useful in confirming significant heart disease in the presence of a heart murmur in dogs with suspected MMVD. Both biomarkers tend to increase with the progression of MMVD (5, 6). However, there is currently no clear cutoff for either biomarker that defines a particular stage of the disease, such as stage B2, at which point the dog may benefit from therapy (7). One study found that an NT-proBNP concentration greater than 1,100 pmol/L identified stage B2 but this was associated with a low sensitivity of 56% (specificity 85%). However, when various physical examinations and laboratory variables were included in a model, the accuracy of identifying stage B2 improved (8).

To capture the various pathophysiological processes involved in MMVD and its progression to congestive heart failure, there is a need for additional tools that can reliably diagnose and stage canine MMVD. A promising approach is the application of microRNA (miRNA) profiling. MicroRNAs are small, non-coding RNA molecules that regulate gene expression and are specifically linked to numerous biological and pathological processes, making them ideal biomarkers. Advantageously, miRNAs can be stored for several days at room temperature or for several years at −20°C with minimal degradation (9, 10). Found in cardiac tissue (11) and circulating in plasma within extracellular vesicles or associated with proteins (e.g., AGO2) (12), they have received increasing recognition as potential diagnostic tests in veterinary cardiology (13). However, limitations in their use as CBs exist as they have no standardized units of measurement, and a plethora of different miRNAs are linked to MMVD in dogs (14–18), indicating that no single marker can be used as a gold standard in isolation. Indeed, a panel of different miRNAs that are upregulated or downregulated depending on the presence of the stage of MMVD and its progression may be advantageous.

The objectives of this study were two-fold: preliminary assessment of an integrated approach analyzing the expression pattern of 15 miRNA markers by customized predictive classification modeling using a miRNA panel to discriminate MMVD patients from healthy controls; and assessment of the same method for finer-grained differentiation between the pre-clinical (stage B) and clinical (stage C/D) MMVD patients.

## 2 Materials and methods

### 2.1 Institutional animal care and use committee or other approval declaration

The use of surplus case and control samples in the study from the Cardiology Service of the Small Animal Teaching Hospital was approved by the University of Liverpool ethics review (VREC851). The use of surplus control samples in the study was considered by Scotland’s Rural College (SRUC) Ethical Committee, and permission was granted as all samples had consent for research use of surplus at the time of submission to SRUC Veterinary Services.

### 2.2 Sample collection and inclusion/exclusion criteria

A total of 123 canine blood samples (serum or plasma) were collected from unique cases by the cardiology clinic at Center 1 (Cardiology Service of the Small Animal Teaching Hospital, University of Liverpool, Liverpool, UK) and the diagnostic laboratory at Center 2 (SRUC Veterinary Services, SRUC, Edinburgh, UK) between September 2020 and December 2021. Blood samples were surplus from clinically indicated testing, and hence were not collected specifically for this study. After collection in either plain blood tubes for serum or EDTA tubes for plasma, the samples were centrifuged, and the serum or plasma was decanted into plain tubes and stored at −20°C for up to 1 month before shipping on dry-ice to the laboratory for miRNA analysis. The samples were stored at −20°C until analysis, for a maximum period of 9 months. Additional data collected at sampling included breed, age, sex, and neuter status for all samples. The only inclusion criterion for the MMVD group was the confirmed presence of the condition. Cases of MMVD with a history or clinical signs of concurrent disease were excluded from the study. For the control group, the inclusion criteria were good general health based on laboratory testing (e.g., pre-anesthesia screening) and the absence of the typical murmur associated with mitral regurgitation. Any control dog with significant concurrent disease was excluded.

All MMVD cases (*n* = 73) were sourced from Center 1 and confirmed with echocardiography. Cases were categorized into pre-clinical (stage B1/B2) and clinical (stage C/D) using the American College of Veterinary Internal Medicine (ACVIM) guideline definitions (3). The concentrations of current cardiac blood biomarkers (NT-proBNP and cTnI) were also available for some cases. The second-generation NT-proBNP assay was used (IDEXX, Wetherby, UK). Troponin I was either assayed using IMMULITE 2000 (Siemens; IMM) or the Beckman Coulter Access high sensitivity assay (IDEXX, Wetherby, UK; HS).

Control cases from Center 1 were subject to the same examinations for selection (*n* = 18) and did not have any heart murmur identified. Those from Center 2 were selected based on having no known current pathology; no heart murmur reported and normal biochemistry and hematology parameters at the time of submission (*n* = 32). The results of clinical pathology testing were reviewed to assess general health and to identify any significant comorbidity and exclusion criteria.

### 2.3 Clinical examination, auscultation, and echocardiography of MMVD cases

Physical examination findings were recorded at the time of examination by one of the authors, and the following data were retrieved: heart rate and rhythm, respiratory rate, and cardiac and pulmonary auscultation findings. The presence, point of maximal intensity, and grade (out of six) of any heart murmur were recorded, noting radiation. Systolic blood pressure was measured routinely in all cases using Doppler sphygmomanometry (19). Echocardiography with a GE Vivid E95 machine and a 6S 2.7–8.0 MHz phased-array transducer was performed on each dog within an hour after auscultation by the auscultating clinician. Cases were assessed by a cardiology diplomate or a cardiology resident under the direct supervision of a diplomate. The diagnosis of MMVD was made based on the typical nodular thickening of the mitral valve, associated with mitral regurgitation, as noted on color flow Doppler echocardiography (3). A complete Doppler echocardiographic assessment was carried out, but data retrieved to allocate the ACVIM stage (3) for this study included the right parasternal 2D short-axis left atrium to aortic (LA/Ao) ratio, with an optimized left atrial diameter, including aortic valves, the first frame after they closed (in early diastole) (20) (LA/Ao). Left ventricular M-mode was obtained with the M-mode cursor positioned on right parasternal short axis views at the level of the papillary muscle tips, to bisect the left ventricular cavity. The left ventricular internal diameter in diastole (LVIDdN) was normalized for body weight by allometric scaling (21). Fractional shortening was calculated from M-mode (FS) as a percentage. In addition, as a further assessment of left atrial size, from right parasternal long axis 4 and 5 chamber views, optimizing the left atrium and the aorta, respectively, the maximal left atrial diameter (LAmax) at the end of ventricular systole and the diameter of the aortic annulus between open aortic valve leaflets were measured, and the LAmax/aortic annulus ratio calculated (22). From right parasternal long-axis views optimizing the left ventricular length and area, Simpson’s method of disks (SMODs) was used to determine the end-diastolic and end-systolic left ventricular volumes (EDV and ESV, respectively; mLs). The ejection fraction percentage was calculated as [(EDV-ESV)/EDV] × 100. The EDV and ESV were normalized for body weight (23). From the left apical 4 chamber view, aligned for transmitral flow, spectral Doppler was obtained, and the mitral E wave velocity (Ev) was measured. From the apical 5 chamber view, with the sample volume between transmitral flow and left ventricular outflow tract (LVOT) flow, spectral Doppler was obtained, and the isovolumic relaxation time (IVRT) measured at the end of LVOT flow and start of the mitral E wave. The E/IVRT was measured as an estimate of left-sided filling pressures (24). From the LVIDdN, FS, LA/Ao, and Ev, the Mitral INsufficiency Echocardiographic (MINE) score was calculated as previously described (25). Where thoracic radiographs were obtained, the vertebral heart size was measured from the right lateral view (26), and any cardiogenic pulmonary edema was noted. The medications that dogs were receiving at the time of assessment were documented, but the medication subsequently prescribed is not reported.

### 2.4 MicroRNA expression profiling

The heart disease-specific miRNA panel was selected through a review of manuscripts identified in a PubMed-based literature search encompassing research on cardiac and mitral valve disease in humans, dogs, cats, and rodents (Supplementary Table 1). This was supplemented and adjusted using the mirPath v3 database (27) to predict the likely roles of microRNAs based on the expected pathology and disease pathways. This identified 20 miRNAs noted to have altered expression during heart pathology, which was mapped to miRBase (release 22.1) for the confirmation of sequences and nomenclature (27) (Table 1). The most stable expressed miRNAs in an exploratory dataset of canine and feline samples (*n* = 556) were selected as normalizer miRNAs using the geNorm function of Fireplex Analysis Workbench 2.0.274 (28) (Abcam, Cambridge, UK). These included miRNAs previously suggested to be involved in canine cardiac disease but were found to have low variance in our dataset. Finally, three off-species (i.e., non-mammalian) miRNAs were also included to act as background controls (oan-miR-7417-5p, cel-mir-70-3p, and ath-mir167d). These provided a custom 23-plex panel for the Fireplex miRNA platform (Abcam, Cambridge, UK). For expression profiling, 50 μl of aliquots of sera or plasma from each sample were incubated with Fireplex capture microbead particles specific to the miRNA targets and processed following the manufacturer’s instructions with optimized hybridization, melt-off and capture temperatures of 39, 62, and 39°C, respectively. The mean fluorescence intensities (MFIs) of miRNA-specific particles per sample were measured to quantify miRNA expression using a NovoCyte flow cytometer and Novosampler Pro software (Agilent, Santa Clara, USA). Raw data were exported to Fireplex Analysis Workbench 2.0.274 (Abcam, Cambridge, UK), where normalized expression values were prepared using the ‘geomean’ function with the pre-selected normalizers identified in the preliminary dataset.

**Table 1:** Summary information for the profiling panel indicating mature sequence and descriptive references.

miRNA	Mature sequence	References
cfa-miR-30b-5p	UGUAAACAUCCUACACUCAGCU	(14–16)
cfa-miR-30d-5p	UGUAAACAUCCCCGACUGGAAGCU	(11)
cfa-miR-128-3p	UCACAGUGAACCGGUCUCUUU	(14, 17)
cfa-miR-133a-3p	UUGGUCCCCUUCAACCAGCUGU	(60, 61)
cfa-miR-133b-3p	UUUGGUCCCCUUCAACCAGCUA	(16)
cfa-miR-142-5p	CCCAUAAAGUAGAAAGCACUA	(17)
cfa-miR-206-3p	UGGAAUGUAAGGAAGUGUGUGG	(12, 18)
cfa-miR-320-3p	AAAAGCUGGGUUGAGAGGGCGA	(62)
cfa-miR-423a-5p	UGAGGGGCAGAGAGCGAGACUUU	(17)
cfa-miR-499-5p	UUAAGACUUGCAGUGAUGUUU	(63)
cfa-let-7b-5p	UGAGGUAGUAGGUUGUGUGGUU	(47, 64)
cfa-let-7e-5p	UGAGGUAGGAGGUUGUAUAGUU	(17)
hsa-let-7i-5p	UGAGGUAGUAGUUUGUGCUGUU	(65)
hsa-miR-29a-3p	UAGCACCAUCUGAAAUCGGUUA	(66)
hsa-miR-486-5p	UCCUGUACUGAGCUGCCCCGAG	(67)
cfa-miR-17-5p*	CAAAGUGCUUACAGUGCAGGUAG	(11)
cfa-miR-130b-3p*	CAGUGCAAUGAUGAAAGGGCAU	(47)
cfa-miR-20a-5p*	UAAAGUGCUUAUAGUGCAGGUAG	(11)
cfa-miR-23a-3p*	AUCACAUUGCCAGGGAUUU	(68)
cfa-miR-26a-5p*	UUCAAGUAAUCCAGGAUAGGCU	(69)

Normalizer miRNAs are indicated with * (see Section 2.4).

### 2.5 Data preparation and exploration

The Fireplex-processed dataset consisted of 97 miRNA expression profiles issued from 50 controls and 47 MMVD cases. MMVD cases were further divided into 29 pre-clinical and 18 clinical MMVD cases. Data were generated in two batches of 48 and 49 samples, respectively. Each miRNA profile was formed by measuring the normalized MFI of 15 miRNAs common across the two data batches. For each miRNA profile, MFIs were standardized by a centered log-ratio transformation applied to each sample to handle the compositionality of the quantification of miRNA molecules derived from varying sequencing library sizes across samples (29, 30). To manage variation due to batch effects, the weighted PLS-DA-batch correction method was applied (31). This method was specifically designed for an unbalanced batch x disease status setting.

Three datasets were generated:

1. The first included all 97 samples to analyze characterization and discrimination between control and MMVD samples.
2. The second included only the MMVD samples and was used to explore potential patterns that could differentiate between the pre-clinical and clinical MMVD cases.
3. A final dataset consisting of controls only was used to assess the potential confounding effects of using both plasma and sera samples.

The first two datasets were illustrated by heatmaps and approximately represented in two dimensions using principal component analysis (PCA) (32) to allow the initial exploration and identification of miRNA patterns. The first two principal components, accounting for the largest fraction of the original data variability, were used to produce a planar biplot display where samples and miRNA signals were jointly represented by points and rays from the origin, respectively (with the rays indicating directions of increasing miRNA expression relative to the others). Potential differences in miRNA profiles according to the type of sample were explored by applying PCA to the third dataset. Furthermore, the existence of a relevant grouping structure here was assessed using data clustering methods. Thus, both the popular *k*-means algorithm and hierarchical clustering were applied (33), and the optimal number of clusters in the data was determined by computing the Gap statistic (34).

### 2.6 Predictive classification modeling

After preliminary investigation and comparative assessment of alternative statistical and machine learning approaches to select an optimal predictive modeling formulation, penalized logistic regression (PLR) models (33, 35) were fitted to the processed miRNA signals for predictive classification of samples into the following:

1. MMVD cases or controls.
2. Pre-clinical MMVD or clinical MMVD.

Formally, given a 2-class response variable y that takes values of 1 (positive status) with probability p and 0 (negative status) with probability 1−p , and a vector of k=15 processed miRNA signals (mean-centered) acting as predictors $x=\left(x_{1},\ldots ,x_{k}\right)$ , a logistic regression model of the form


$$\ln\frac{p}{1-p}=\beta_{0}+\overset{k}{\underset{j=1}{\sum }}\beta_{j}x_{j}$$

was established. Using the maximum likelihood estimation method, the PLR model coefficient estimates $\left({\hat{\beta }}_{0},{\hat{\beta }}_{1},\ldots {\hat{\beta }}_{k}\right)$ were obtained by maximizing the penalized log-likelihood function:


$$\max\left\{\overset{n}{\underset{i=1}{\sum }}\left[y_{i}\left(\beta_{0}+\overset{k}{\underset{j=1}{\sum }}\beta_{j}x_{ij}\right)-\ln\left(1+e^{\beta_{0}+\overset{k}{\underset{j=1}{\sum }}\beta_{j}x_{ij}}\right)\right]-\lambda \overset{k}{\underset{j=1}{\sum }}{\left|\beta_{j}\right|}^{2}\right\}\text{,}$$

where n refers to the number of samples and λ is the penalty parameter, so that the coefficients less contributing to the prediction of the outcome were shrunk toward zero. Note that this formulation, including a quadratic penalty, corresponds to so-called L_2_ regularization or ridge regression. Such a penalty aids in preventing overfitting, favoring model unbiasedness, sparsity, and a stable fit with large numbers of predictors, typically affected by multicollinearity. Given the model estimates, the predicted status probabilities for a sample were obtained using


$$\hat{p}=\frac{1}{1+e^{-\left({\hat{\beta }}_{0}+{\hat{\beta }}_{1}x_{1}+\ldots +{\hat{\beta }}_{k}x_{k}\right)}}\text{,}$$

with a sample being allocated the status with the highest probability. Thus, in the MMVD group against the control setting, a sample was diagnosed positive when $\hat{p}>.5$ and negative otherwise. This default threshold was regarded optimal after a joint assessment of trade-offs between model performance metrics over a range of decision thresholds in [0.1, 0.9] (not shown). In comparing pre-clinical MMVD to clinical MMVD, the latter was considered the positive status. Tuning to determine the level of shrinkage λ , parameter fitting by maximum likelihood, and model performance assessment were all embedded into a 5-time repeated 10-fold cross-validation (CV) pipeline. That is, the input data were randomly partitioned into 10 folds, where sequentially nine folds were used to train the model and one fold (blind to the trained model) was used to test its performance, with this randomization being repeated 5 times. Such random and repeated splitting into train and test sets enables a fairer and more robust assessment of the performance of the method in a real-world setting [see, e.g. (33), for further details]. As the number of samples in each class was unbalanced in the input data, particularly when confronting pre-clinical MMVD and clinical MMVD, 29 and 18 samples, respectively, the minority class was over-sampled within the CV runs by using the synthetic minority sampling technique (SMOTE) (36), aiming to minimize the effect of this issue on model performance assessment. Note that, along with the miRNA signals, it is feasible to include other variables as predictors in the models to assess their potential to improve performance in sample classification. Thus, the sex of the animals was initially considered in the PLR models as a potential predictor along with the miRNA signals, but the associated model coefficients $\hat{\beta }$ resulted in not being statistically significant at the usual 5% significance level and it was omitted from the final modeling. Performance metrics included overall accuracy, the area under the receiver operating curve (AUC-ROC), sensitivity, specificity, and the F1 score (regarded as a fairer assessment of accuracy in case of unbalanced classes) (33). All these metrics ranged in [0, 1], with values closer to 1 indicating better performance. They were measured against each validation set and averaged across CV runs to assess how the model might perform when asked to predict from independent blind samples. These measures were accompanied by 95% confidence intervals (CIs) where available. Moreover, Cohen’s Kappa metric was used to compare the accuracy of the proposed model against a naïve classifier (based on random guessing), and the significance of the increased accuracy relative to the no information rate) was statistically tested (37). All data analysis and modeling were conducted on the R system for statistical computing v4.2.1 (38), using routines implemented in the specialized packages caret, MLeval, and pROC for model training and assessment (39–41).

## 3 Results

### 3.1 Description and classification of the cohort population

**Figure 1:**
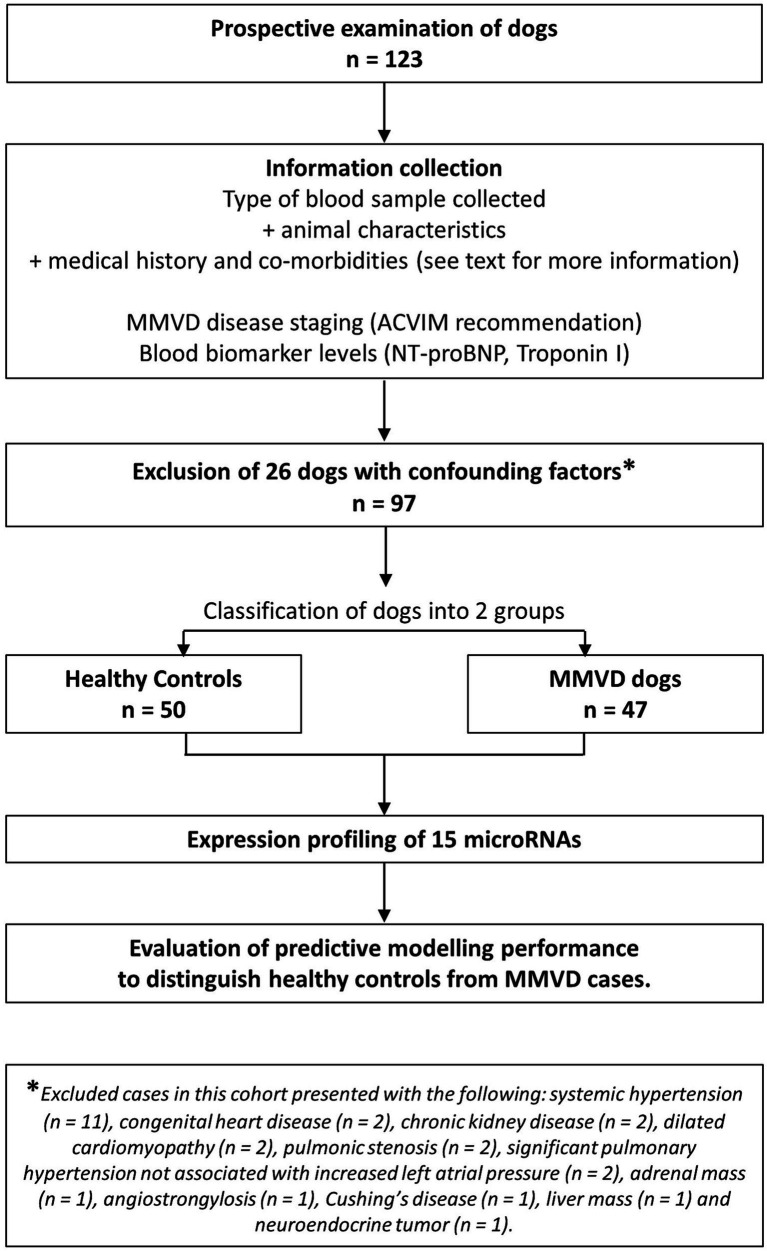
Flow diagram describing the clinical cohort recruited to the study.

Of the 123 dogs enrolled in the study, 26 were excluded because of clinically important systemic or other organ-related diseases (Figure 1).

The 97 dogs included in the study were first divided into two groups: MMVD cases (*n* = 47) and controls (*n* = 50) (Table 2). Within the 47 MMVD cases, 21 dogs were stage B1 (44.7%), 8 dogs were stage B2 (17%), 8 dogs were stage C (17%), and 10 dogs were stage D (21.3%).

**Table 2:** Characteristics of 97 dogs recruited for the study.

	Healthy controls (*n* = 50)	MMVD dogs (*n* = 47)
Age, median [range]	4 [2–15]	10 [6.1–13.8]
Sample type, number (%)		
Serum	26 (52)	46 (97.9)
Plasma	24 (48)	1 (2.1)
Sex, number (%)		
F	26 (52)	7 (14.9)
FN	9 (18)	15 (31.9)
M	10 (20)	9 (19.1)
MN	5 (10)	16 (34)
Breed, number (%)		
Chihuahua	1 (2)	3 (6.4)
CKCS	2 (4)	19 (40.4)
Cocker Spaniel	4 (8)	2 (4.3)
Dobermann	3 (6)	1 (2.1)
German Shepherd	3 (6)	0 (0)
Great Dane	15 (30)	0 (0)
Jack Russell Terrier	2 (4)	4 (8.5)
Labrador	4 (8)	1 (2.1)
Other pure breeds	15 (30)	9 (19.2)
Mixed Breed	1 (2)	8 (17)

CKCS, Cavalier King Charles Spaniel; F, female; FN, female neutered; M, male, MN, male neutered.

The type of blood sample collected, along with the age, sex, and breed of each animal, were recorded (Table 2). Of the control samples, 52% were sera and 48% were plasma, with 97.9% sera and 2.1% plasma in MMVD samples. However, we found no evidence for sub-structuring in plasma vs. serum samples when the data were represented in two dimensions using PCA or when seeking to identify clusters of miRNA profile data within the control group using both *k*-means and hierarchical clustering algorithms, with the most likely number of clusters being in fact one according to the associated Gap statistic. This suggests no meaningful influence of sample type in our study (data not shown).

### 3.2 NT-proBNP and troponin I levels

At least one CB (NT-proBNP or cTnI) was available for a proportion of the controls (*n* = 17) and MMVD cases (*n* = 27). The low number of available results meant statistical comparison to the presented miRNA profiling was not possible; however, CB results and the percentage of these cases correctly classified by miRNA profiling are provided (Table 3).

**Table 3:** MMVD classification and blood biomarker levels in cases where available.

Stage	Healthy controls	MMVD dogs	
	NA	Pre-clinical (B1/B2)	Clinical (C/D)
NT-proBNP			
Number of dogs with available data	15	14	0
Conc (pmol/L) Median (min–max) (upper ref. value: 900 pmol/L)	258 (250–840)	737 (250–1,575)	NA
% correctly classified, where clinical significance indicated at >900 pmol/L	100%	36%	NA
Troponin I			
Number of dogs with available data (HS)	13	2	0
Number of dogs with available data (IMM)	4	11	7
Conc (ng/ml) Median (min–max) (upper reference value: >0.07 ng/ml HS)	0.01 (0.01–0.06)	0.04 (0.03–0.05)	NA
Conc (ng/ml) Median (min–max) (upper reference value: >0.15 ng/ml IMM)	0.03 (0.01–0.16)	0.10 (0.03–0.45)	0.31 (0.13–2.63)
% correctly classified, clinical significance indicated at >0.07 ng/ml (HS) and > 0.15 ng/ml (IMM)	94%	23%	86%
Number of dogs with NT-proBNP and/or cTnI available	17	20	7
% correctly classified with miRNA profiling	88%	95%	100%

HS, High sensitivity troponin I concentration; IMM, IMMULITE troponin I assay; NA, not applicable.

### 3.3 Predictive classification of healthy controls and MMVD cases

**Figure 2:**
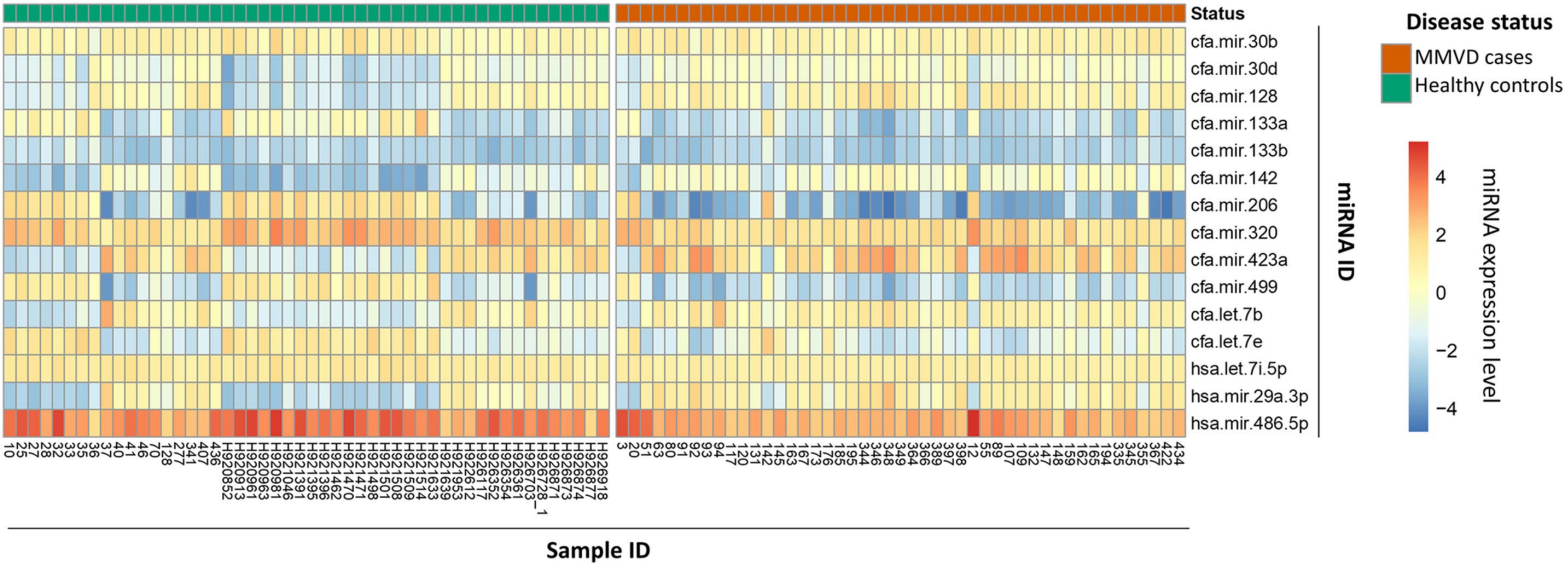
MicroRNA expression profile heatmap of healthy controls and MMVD cases. Heatmap representation of 15 miRNA expression profiles in 97 dog blood samples divided into two groups according to their disease status, healthy controls (left side; green in top row) or MMVD patients (right side; red in top row). MicroRNA expression is represented as a color gradient based on mean fluorescence, increasing from bottom to top. The samples and miRNA identification numbers are shown in the bottom *X*-axis, and right-hand side *Y*-axis, respectively.

The miRNA expression profile of each of the 97 dogs is represented as a heatmap (Figure 2), distinguishing between controls and MMVD cases. The overall numerically highest expression was observed for miRNA hsa-mir486-5p, particularly in the control group, agreeing with the typically high abundance of this miRNA in peripheral blood (42). The numerically lowest expression across all samples was observed for miRNA cfa-mir-206-3p, particularly for the MMVD samples.

**Figure 3:**
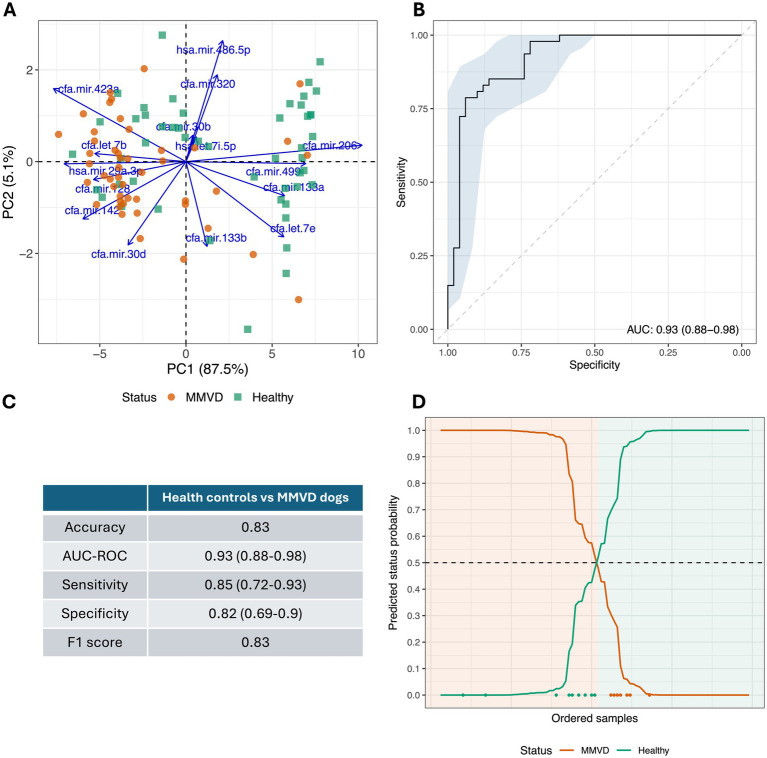
Cross-validated diagnostic performance of the predictive model to distinguish healthy controls from MMVD cases. **(A)** Principal component analysis (PCA) biplot of processed miRNA profiles of all 97 collected samples that distinguish healthy controls from MMVD dogs. **(B)** Cross-validated receiver operating curve (ROC) for healthy controls vs. MMVD dogs (combining stage B1/B2 and stage C/D MMVD) is shown. Area under the curve (AUC) and confidence interval (95% CI) are indicated at the bottom right of the diagram. **(C)** Summary cross-validated performance statistics from predictive model to distinguish healthy controls from MMVD dogs (95% CI showed in parenthesis where available). **(D)** Predicted status probabilities for each collected sample. Samples are ordered from left to right on the *X*-axis according to their probability. Classification is based on a usual 0.5 probability threshold (dotted line), background color indicates actual dog disease status. Misclassified samples are indicated at the bottom of the diagram using green and orange dots in accordance with their disease status.

A performance summary of the prediction model distinguishing between controls and MMVD dogs is provided in Figure 3. The PCA biplot of the entire miRNA dataset based on the first two principal components (PC1 and PC2) demonstrates that variability between MMVD and controls was mostly represented along the first PC axis (PC1; 87.5% variation explained; Figure 3A). Variation along PC1 is related to the differential expression of a small number of miRNAs, including cfa-mir-206-3p, the most highly expressed miRNA in the control group and hsa-mir-29a-3p, the most highly expressed miRNA in the MMVD group. Other miRNAs, such as cfa-mir-133b-3p, contributed to the variation in PC2 and were poorly associated with MMVD. Receiver operator characteristic analysis of the model provided an area under the curve (AUC) value of 0.93 (0.88–0.98; Figure 3B), with an overall cross-validated accuracy of 0.83, a sensitivity of 0.85 (0.72–0.93), a specificity of 0.82 (0.69–0.90), and an F1 score of 0.83 (Figure 3C). Misclassification of samples was more frequent for controls and occurred more often in samples assigned class probabilities close to 0.5 by the PLR model (Figure 3D). Moreover, a Kappa metric value of 0.70 supported that our classifier performed substantially better than a naïve classifier, with an accuracy statistically significantly higher than the no information rate (NIR = 0.51, *p* < 0.001).

### 3.4 Predictive classification of pre-clinical (stage B) and clinical (stage C/D) MMVD cases

**Figure 4:**
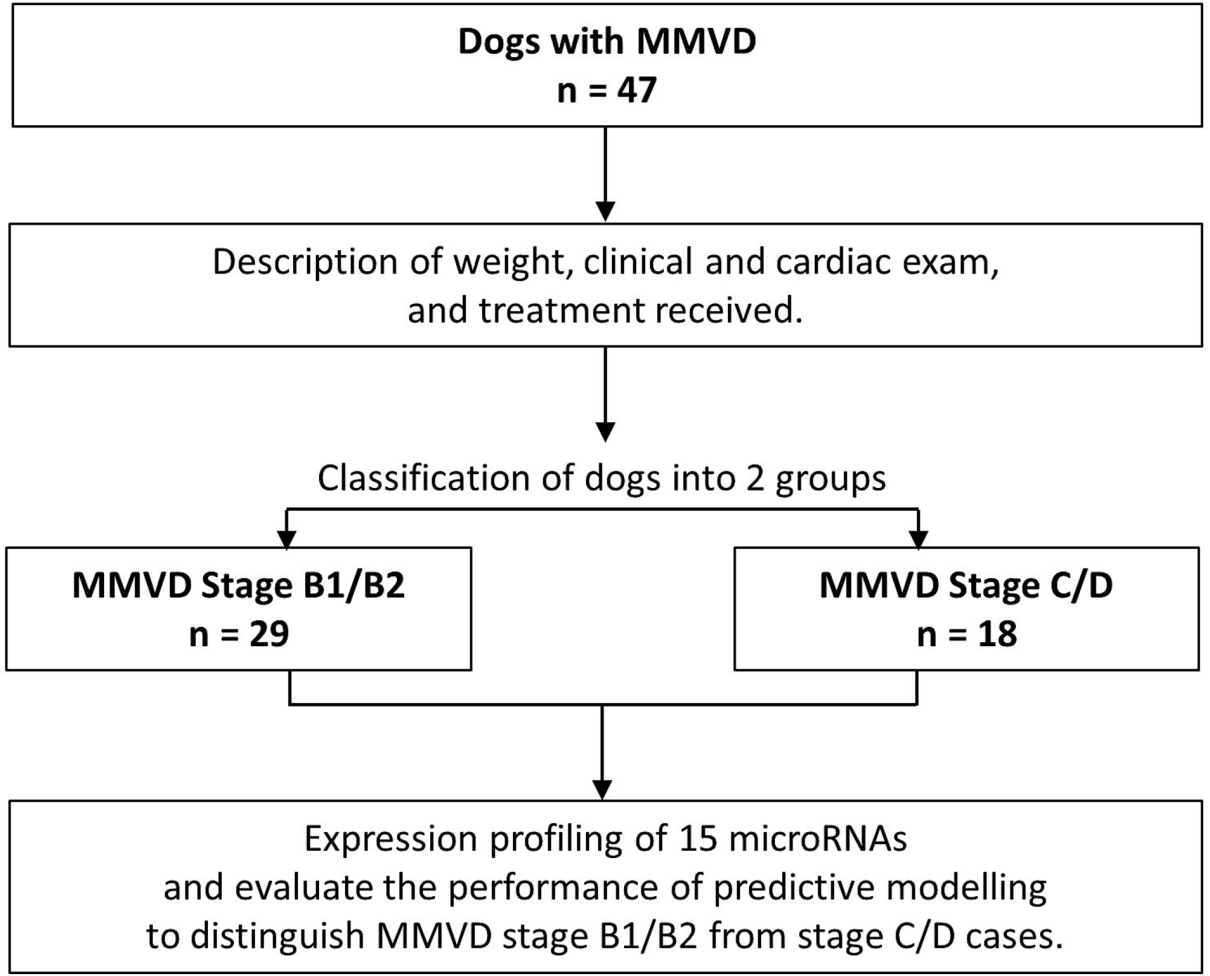
Flow diagram further describing the MMVD clinical cohort recruited to the study.

The 47 MMVD dogs were subdivided into pre-clinical and clinical groups (Figure 4). A total of 29 pre-clinical cases and 18 clinical cases were identified (Table 4). Clinical examination and echocardiographic findings of the pre-clinical and clinical MMVD groups are summarized in Table 4. In keeping with clinical expectation, all echocardiographic measurements taken to evaluate cardiac size were numerically higher in clinical than pre-clinical cases. In accordance with disease severity, significantly more dogs were receiving cardiac medications at presentation in the clinical group.

**Table 4:** Weights, clinical and cardiac exams, and medication of 47 MMVD dogs recruited for the study.

	MMVD	
	Stage B1/B2 (*n* = 29)	Stage C/D (*n* = 18)
Physical exam: median [range]		
Weight (kg)	9.8 [3.4–42]	8 [2.6–31.5]
Clinical exam: median (IQR)		
Respiratory rate	30 (26–36)	36 (28–44)
Heart rate	120 (100–138)	135 (120–150)
Heart murmur grade (/6)	3 (2–4)	5 (5–5)
SBP (mmHg)	147.5 (133.8–156.5)	140 (125–152.5)
Cardiac measurements and function: median (IQR)		
VHS (RL thoracic radiograph)	10 (9.8–11.2)	12.5 (12.3–13.1)
LVIDdN (M-mode)	1.5 (1.4–1.6)	2.1 (1.8–2.2)
LVIDsN (M-mode)	0.9 (0.8–1)	1 (0.8–1.2)
FS % (M-mode)	41 (35–45)	47 (43.2–56.8)
EDV (mLs) (SMOD)	25 (19–39)	35 (23.5–64.5)
ESV (mLs) (SMOD)	8 (5–13)	7.5 (4.3–22.8)
EDV/kg	2.3 (1.9–2.8)	4.5 (3.6–5.6)
ESV/kg	0.8 (0.6–1)	1.1 (0.8–1.4)
EF % (SMOD)	63.5 (59.5–68.7)	72.5 (66.5–80.8)
LAmax (cm)	3.3 (3–4)	4.5 (3.8–5)
LAmax/Ao annulus systole	2.6 (2.2–3.1)	3.6 (3.5–4.2)
LA/Ao diastole	1.5 (1.4–1.7)	2.2 (2–2.4)
Mitral E (m/s)	0.7 (0.6–0.9)	1.1 (0.8–1.4)
Mitral E/IVRT	1.2 (0.8–1.4)	2.2 (1.4–3)
MINE score	5 (4–9)	9 (4–13)
Treatment received at time of initial assessment: number (%)		
Pimobendan	5 (17.2)	16 (88.9)
Benazepril	2 (6.9)	16 (88.9)
Spironolactone	2 (6.9)	16 (88.9)
Furosemide/Torsemide	0 (0)	17 (94.4)
Amlodipine	0 (0)	1 (5.6)
Sotalol	0 (0)	1 (5.6)

Ao, aorta; EDV, end-diastolic volume; E, early diastolic transmitral flow wave velocity; EF, ejection fraction; ESV, end-systolic volume; FS, fractional shortening; IVRT, isovolumic relaxation time; LAmax, left atrial maximal diameter at end of systole (right parasternal 4 chamber view); LA/Ao, short-axis early diastolic diameter ratio of left atrium and aorta; LVIDdN, left ventricular internal diameter; diastole; normalized for body weight; LVIDsN, left ventricular internal diameter; systole; normalized for body weight; SBP, systolic blood pressure; SMOD, Simpson’s method of discs; VHS, vertebral heart score.

**Figure 5:**
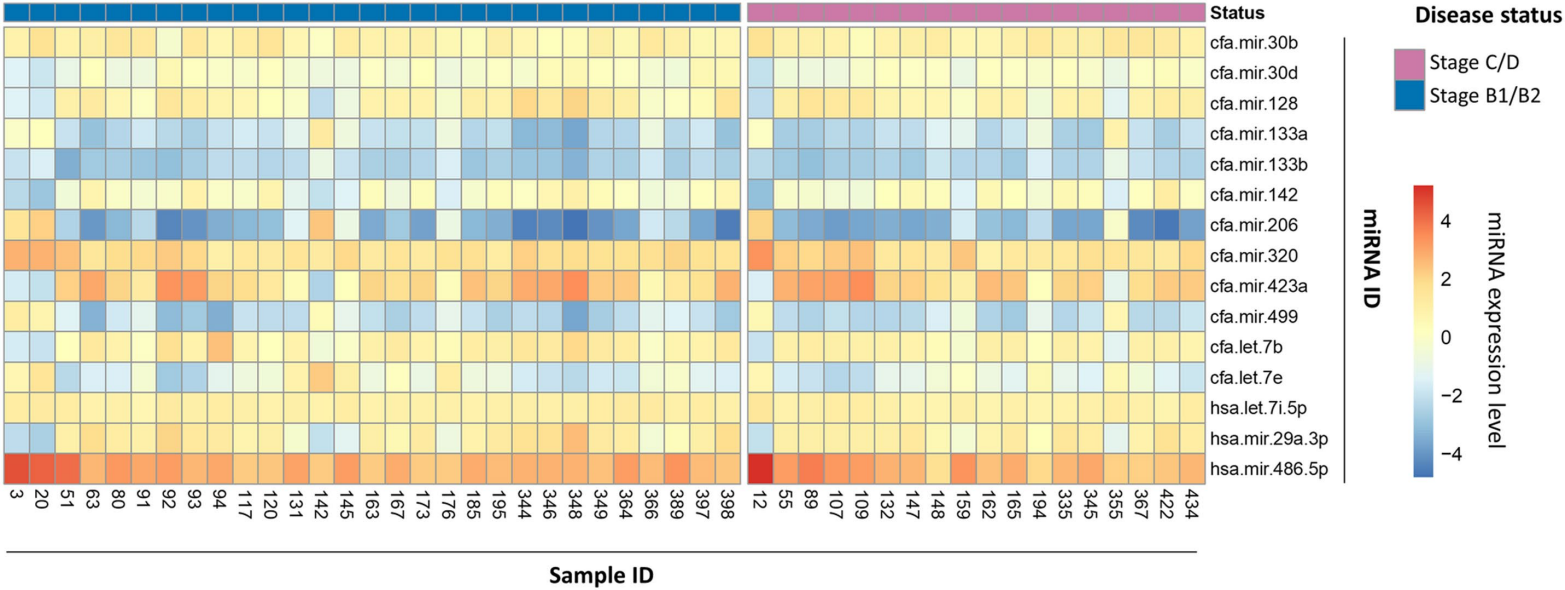
MicroRNA expression profile heatmap of stage B1/B2 and stage C/D MMVD cases. Heatmap representation of 15 miRNA expression profiles in 47 dog blood samples classified into two groups according to their MMVD disease stage: stage B1/B2 (blue top row, on left) and stage C/D (purple top row, on right). Classification is based on clinical and cardiac examinations, following ACVIM guidelines. MicroRNA expression is represented as a color gradient based on mean fluorescence, increasing from bottom to top. The samples and miRNA identification numbers are shown in the bottom *X*-axis and right-hand side *Y*-axis, respectively.

**Figure 6:**
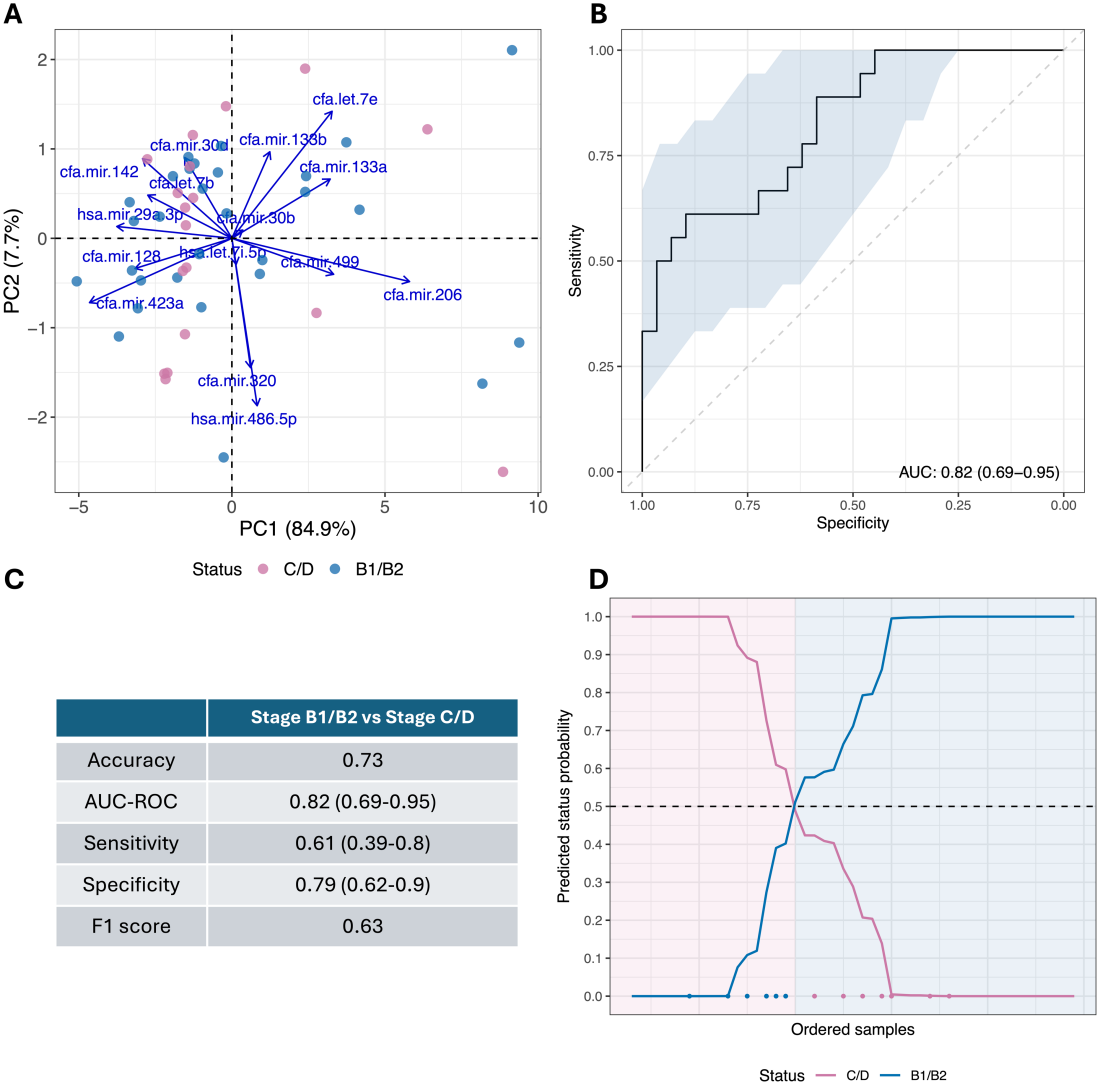
Cross-validated diagnostic performance of the predictive model to distinguish stage B1/B2 from stage C/D MMVD dogs. **(A)** Principal component analysis (PCA) biplot of processed miRNA profiles of 47 collected samples that distinguish stage B1/B2 from stage C/D MMVD dogs. **(B)** Cross-validated receiver operating curve (ROC) for stage B1/B2 MMVD dogs vs. stage C/D MMVD dogs is shown. Area under the curve (AUC) and confidence interval (95% CI) are indicated at the bottom right of the diagram. **(C)** Summary cross-validated performance statistics from predictive model to distinguish stage B1/B2 from stage C/D MMVD dogs (95% CI indicated in parenthesis where available). **(D)** Predicted status probabilities for each collected sample. Samples are ordered from left to right on the *X*-axis according to their probability. Classification is based on a usual 0.5 probability threshold (dotted line), background color indicates actual dog disease status. Misclassified samples are indicated at the bottom of the diagram using blue and pink dots in accordance with their disease status.

The miRNA expression profile of each of the 47 MMVD dogs is represented as a heatmap (Figure 5), distinguishing between the pre-clinical and clinical MMVD cases. As seen previously (Figure 2), the numerically highest expression was observed for miRNA hsa-mir486-5p, and the numerically lowest expression was observed for miRNA cfa-mir-206-3p; however, differentiation between the pre-clinical and clinical MMVD samples was not as distinct, and groups are not clearly distinguished in the PCA biplot (Figure 6A). The ROC analysis provided a moderately high AUC value of 0.82 (0.69–0.95), a sensitivity of 0.61 (0.39–0.80), and a specificity of 0.79 (0.62–0.90; Figures 6B,C). The F1 score was a moderate 0.63 in this case. The overall cross-validated accuracy was 0.73, with misclassification being similar for both groups (Figure 6D). The corresponding Kappa metric value of 0.36 implied just a fair improvement relative to the naïve classifier, although still statistically significantly outperforming the no information rate (NIR = 0.62, *p* < 0.001).

## 4 Discussion

The expression profiles of the selected 15 miRNAs in this preliminary study allowed convincing distinction of controls from MMVD cases, with the optimized predictive model providing an AUC value of 0.93, with an overall cross-validated accuracy of 0.83, a sensitivity of 0.85, and a specificity of 0.82. Variability between the MMVD and control samples was mostly related to the differential expression of a small number of miRNAs.

The MMVD group saw hsa-mir-29a-3p most highly expressed, matching observations that mir-29 is upregulated in pathological remodeling via the Wnt signaling pathway and is associated with myocardial fibrosis in murine models (43–45). Other miRNAs, such as cfa-mir-133b-3p, were poorly associated with any group, contradicting observations in Dachshunds, where miR-133b was downregulated in stage C compared to stage A dogs and thus thought to be a possible marker of CHF (16). This discrepancy could be linked to variations in size and the wider breed diversity in the current study population. Differential expression of cfa-mir-206-3p was also apparent, most highly expressed in the control group. Atrial tissue cfa-mir-206-5p increased expression has been associated with tachycardia pacing-induced atrial fibrillation and autonomic nerve remodeling in experimental dogs (46); atrial fibrillation may naturally occur in MMVD due to atrial stretch, although not present in this study’s cases. Other studies reviewing circulating mir-206 have not shown differential expression between cases and controls (18).

It is apparent that different miRNAs behave differently with the stage of the disease (15, 47). For example, miR-30b-5p is upregulated in stage B1 compared to controls in Cavalier King Charles spaniels (CKCS) and then declines with advancing disease (14, 15), but here miR-30b did not show any significant differences with the presence or stage of MMVD. Therefore, this miRNA panel may have advantages over testing a single miRNA in that the panel could potentially give a fingerprint for a specific stage, or within a specific breed. However, more research is required to elucidate these factors in a larger population.

One possible limitation of the study is some structuring in the data due to age. Although efforts were made to obtain a similar population in the control and MMVD groups, the median age of the control group was numerically lower (4 years) than the median age of the MMVD group (10 years), although the age range of both groups does overlap (2 to 15-years-old and 6.1 to 13.8-years-old, respectively). This is largely related to age being an important factor in the development of MMVD (1–3) making population recruitment challenging. However, the possibility of an age-related effect in our data should be considered. Sex has also been reported as an important risk factor in MMVD (48); however, sex was not identified as a significant factor of disease along with the miRNA profile in our modeling.

In comparing miRNA profiles between pre-clinical and clinical MMVD cases, expression allowed some distinction between the two groups. ROC analysis suggested a moderately high AUC value of 0.82, with a sensitivity of 0.61 and a specificity of 0.79. The 95% CI for these metrics showed a marked variability across cross-validation runs, and the overall cross-validated accuracy was 0.73, with misclassification being similar for both groups, and the F1 score was moderate with 0.63. Taken together, these data suggest a higher level of uncertainty in distinguishing between pre-clinical from clinical MMVD cases, compared to the differentiating between control and MMVD cases. The performance of the method would likely be enhanced by an increased library of training samples so that predictive modeling could better capture the subtleties in miRNA profiles, allowing improved discrimination between disease stages.

In the MMVD group, there was a breed over-representation of CKCSs (40.4%). The breed is an important risk factor for MMVD (1, 3) and the potential influence of specific breed miRNA expression profiles cannot be excluded. There have been discordant results reported between Dachshunds and CKCSs for miR-30b; while upregulation is reported in the CKCS with stage B1 (15), it was reported to be downregulated in the Dachshund with stage B (not subdivided), compared to control dogs (16). There is breed variation for NT-proBNP (49–51) and the same is possible for miRNA expression. However, formal statistical assessment and comparison between breeds and miRNA profiles were not possible in this study due to a high diversity of breed types providing a low number of individuals per breed. In the future, studying greater numbers of specific breeds with and without MMVD in different stages will allow a more thorough exploration of breed influence.

Potential limitations of this preliminary study included imbalances in sample types (serum vs. plasma) between the MMVD and control groups as a possible confounding factor. However, statistical analysis indicated no meaningful effect of this factor on our results. In a further potential limitation, the MMVD group was robustly phenotyped. While this is a common practice in clinical studies, excluding dogs with confounding factors such as systemic hypertension and other cardiac diseases may have led to results that appear more accurate than what would be observed in a general practice population, where co-morbidities are often present. Moreover, some dogs were receiving treatment, and CHF (in stages C/D) may have been well compensated. This may impact the miRNA results as well as cardiac biomarker results (if available), as shown by some low MINE scores for stage C/D (25). Additionally, a proportion of the control dogs, which were recruited by Center 2, had not undergone echocardiography by the authors. The information was restricted to the history and physical examination findings reported to Center 2 by each dog’s primary care veterinarian, and health presumed by their unremarkable hematology and biochemistry findings. Conversely, this limitation may have reduced the accuracy of the miRNA panel reported here. Despite the potential influence of confounding factors, the authors did not note any statistical incongruences indicating significant detrimental influence within the sample population. The authors’ future studies with an expanded population will provide further information on any effect of these limitations.

It was not possible to statistically compare miRNA results and conventional CBs since there were only a low number of CB results available within the study. However, sensitivity (85%) and specificity (82%) in discriminating between MMVD cases and controls are comparable, if not improved, when considering the results of analogous studies. For example, a recent study of 105 dogs, which compared multiple CBs in controls (*n* = 36) and MMVD cases (*n* = 69), found that for classifying dogs as stage B2 or greater, NT-proBNP and cTnI demonstrated sensitivities of 80 and 48.6% and specificities of 64.7 and 70.6%, respectively (52). Although CBs have a trend of increasing values with MMVD severity (5, 53), there is no distinct cutoff for a specific stage of the disease (e.g., B2). Since the progression of MMVD through the stages is a continuum, this is likely true for miRNA expression. However, the use of a panel of markers gives rise to the possibility of producing a fingerprint profile at each stage, with the potential to identify individuals or patterns of miRNA expression that may be predictive of impending stage progression. If a future biomarker panel could reliably identify stage B2, this would be a great asset for primary care practice, for making decisions concerning a dog with a heart murmur, where echocardiography is unavailable. It is anticipated that forthcoming serial, longitudinal monitoring of cases will provide further insight into this possibility.

Although limited in scope, a comparison of the diagnostic capability of miRNA profiling to existing NT-proBNP and cTnI biomarkers showed some promise. NT-proBNP is released into the blood circulation in response to myocardial wall stretch, and therefore correlates with the echocardiographic assessment of left-sided volume overload, as a consequence of mitral regurgitation, such as the left atrium to aortic ratio and the left ventricular diastolic diameter or volume (5). The limitations of using NT-proBNP diagnostically include variability between breeds (49–51), the influence on renal function (54), systemic hypertension (55), and day-to-day variability (56). Troponin I is a marker of cardiomyocyte injury and therefore, it is not usually increased in the pre-clinical stages of MMVD (10). It increases in more advanced disease (6, 57) and is of prognostic significance (4). Concentration was shown to correlate with the severity of myocardial fibrosis on subsequent histopathological examination (58). The limitations include increases due to other systemic diseases and day-to-day variability (59).

The miRNA profiling method performed well in correctly classifying controls, showed a possible advantage in the accurate detection of early-stage B1 and B2 cases over the existing biomarkers, and was comparable in stage C and D cases based on our limited cardiac biomarker data (Table 3). The panel of miRNAs examines signaling and remodeling pathways associated with the pathophysiology and progression of MMVD or CHF. In contrast, conventional CBs look at the consequences of these conditions, such as NT-proBNP indicating increased myocardial wall stress and troponin I signifying cardiomyocyte injury. The miRNA panel has the potential to detect earlier stages of diseases or identify cases likely to progress imminently before changes are evident in echocardiographic or CB.

In summary, this study supports the idea that a multiplexing detection assay of blood miRNAs could be a useful diagnostic tool for the identification of MMVD cases in canine populations. Veterinarians can simply diagnose MMVD by auscultating a compatible murmur in an older small breed dog, so miRNA profiling is not proposed as an initial diagnostic test. However, attending veterinarians need to make decisions about the risks to the patient, therapeutic interventions, and prognostication. The authors propose that the miRNA profiling data should facilitate this by fingerprinting the pathophysiological progression of MMVD or remodeling. Additionally, miRNA profiling provides the possibility to differentiate between pre-clinical (stage B1/B2) and clinical MMVD (stage C/D), thereby enhancing early disease diagnosis. If the cost of this technology is comparable to current CBs, it could be beneficial for clinicians in identifying cases requiring further cardiac investigation and monitoring disease progression or response to treatment. This would be particularly valuable in settings where access to specialist cardiology expertise is limited or where costs are prohibitive for the pet owners. The current study would benefit from training on a larger canine cohort that includes a variety of MMVD cases to continually improve the resolution of the probabilistic predictive modeling. This approach would also enable more extensive statistical comparisons with existing CBs. In our view, the early results presented here suggest that microRNA diagnostic technology has promising upcoming potential, not only in the field of veterinary cardiology but also for current and future applications across the broader veterinary sphere.

## Data Availability

The raw data supporting the conclusions of this article will be made available by the authors on request, without undue reservation.
